# Characterization of Mixtures Based on High-Density Polyethylene and Plasticized Starch

**DOI:** 10.3390/polym16213051

**Published:** 2024-10-30

**Authors:** Maria Daniela Stelescu, Ovidiu-Cristian Oprea, Doina Constantinescu, Ludmila Motelica, Anton Ficai, Roxana-Doina Trusca, Maria Sonmez, Dana Florentina Gurau, Mihai Georgescu, Rodica Roxana Constantinescu, Bogdan-Stefan Vasile, Denisa Ficai

**Affiliations:** 1 Division Leather and Footwear Research Institute, National Research & Development Institute for Textiles and Leather, 93 Ion Minulescu St., 031215 Bucharest, Romania; maria.stelescu@icpi.ro (M.D.S.);; 2 Faculty of Chemical Engineering and Biotechnologies, National University of Science and Technology POLITEHNICA Bucharest, 1-7 Polizu St., 011061 Bucharest, Romania; anton.ficai@upb.ro (A.F.);; 3 Academy of Romanian Scientists, 3 Ilfov St., 050044 Bucharest, Romania; ludmila.motelica@upb.ro (L.M.);; 4 S.C. Monofil SRL, Gheorghe Caranfil 5F, Piatra Neamt, 617410 Savinesti, Romania; 5 Research Center for Advanced Materials, Products and Processes, National University of Science and Technology POLITEHNICA Bucharest, 060042 Bucharest, Romania

**Keywords:** plasticized starch, HDPE, montmorillonite, mechanical properties

## Abstract

This paper presents the obtaining and characterization of blends based on high-density polyethylene (HDPE) and plasticized starch. In addition to plasticized starch (28.8% *w*/*w*), the compositions made also contained other ingredients, such as polyethylene-graft-maleic anhydride as a compatibilizer, ethylene propylene terpolymer elastomer, cross-linking agents, and nanoclay. Plasticized starch contains 68.6% *w*/*w* potato starch, 29.4% *w*/*w* glycerin, and 2% *w*/*w* anhydrous citric acid. Blends based on HDPE and plasticized starch were made in a Brabender Plasti-Corder internal mixer at 160 °C, and plates for testing were obtained using the compression method. Thermal analyses indicate an increase in the crystallization degree of the HDPE after the addition of plasticized starch. SEM micrographs indicate that blends are compatibilized, with the plasticized starch being well dispersed as droplets in the HDPE matrix. Samples show high hardness values (62–65° ShD), good tensile strength values (14.88–17.02 N/mm^2^), and Charpy impact strength values (1.08–2.27 kJ/m^2^ on notched samples, and 7.96–20.29 kJ/m^2^ on unnotched samples). After 72 h of water immersion at room temperature, mixtures containing a compatibilizer had a mass variation below 1% and water absorption values below 1.7%. Upon increasing the water immersion temperature to 80 °C, the sample without the compatibilizer showed a mass reduction of −2.23%, indicating the dissolution of the plasticized starch in the water. The samples containing the compatibilizer had a mass variation of max 8.33% and a water absorption of max 5.02%. After toluene immersion for 72 h at room temperature, mass variation was below 8%.

## 1. Introduction

Among polyolefins, the most frequently used at present is polyethylene (PE) [1,2]. According to some studies [3,4], the global PE market size was estimated at USD 114.89–155.18 billion in 2023 and is expected to grow at a CAGR of 4.1–5.0% from 2024 to 2030. PE is obtained through the polymerization of ethylene and is currently used in various areas, such as the packaging industry (plastic bags, membrane, foil, containers, bottles, pallets, etc.), construction (pipes and fittings, rods, extruded plates, shelves, doors, etc.), the food industry (sliding elements, packaging, etc.), the automotive industry, and agriculture, as well as in consumer goods [5,6,7].

There are several types of polyethylene that differentiate depending on density, degree of ramification (cross-linking), molecular mass, composition, additive content, etc. The best-known types are low-density polyethylene (LDPE) and high-density polyethylene (HDPE). Their properties depend greatly on the molecular mass and the crystalline phase percentage [5,7,8].

HDPE has a low degree of ramification and a high crystalline phase percentage. It has high tensile strength, excellent chemical resistance, is resistant to acids, bases, and oxides, has a high resistance to wear, is non-toxic and odorless, has high resistance to compression and bending, and is a good electrical insulator. It is also easily processed into finished products by methods specific to plastic materials such as extrusion, injection molding, blow molding, and compression molding [8,9].

The main disadvantage of PE is that it is not biodegradable and, as a result, the use of increasingly higher amounts of polyethylene has led to difficulties in waste management [2]. At the same time, through exposure to the action of waves, wind abrasion, ultraviolet radiation from sunlight, etc., it can break down, leading to microplastics. Some studies [10,11,12,13] have shown that these microplastics are not biodegradable and can be harmful to the environment and animal health. For these reasons, the aim is currently both to reduce the amount of plastic and to obtain biodegradable plastic materials [13,14,15]. To reduce the amount of plastic, the most ecological method is mechanical recycling with obtaining mechanically recycled plastic materials [16]. They generally have poorer mechanical properties, because they may contain a number of other ingredients (contaminants) [14]. Due to its benefits, this method has developed a lot in recent years as a result of national and international regulations [15,17]. In a report [18], it is specified that, in Europe in 2022, 7.7 Mt of post-consumer recycled plastics were obtained, and the amount of recycled plastics consumed in the creation of new products was 6.8 Mt (of which 37.5% was used in agriculture, 22.7% for building and construction, 9.7% for packaging, and 4.6% for automotive and other purposes).

Other studies [2,19,20] aimed to improve the biodegradation properties of PE by mixing it with a biodegradable polymer, the most-used being starch. Starch is a polysaccharide found in the seeds, fruits, and tubers of plants. It can be obtained from potatoes, corn, rice, wheat, etc. [21]. Due to the existence of hydrogen bonding interactions and intermolecular forces, it needs to be plasticized so that it can be processed at high temperatures. The main plasticizers used for this purpose are glycerin, sorbitol, and water. In recent years, plasticized starch mixed with other polymers, such as polyethylene, has been used to obtain biodegradable packaging due to its film-forming ability, low cost, renewability, etc. [21,22]. For example, some of the articles made by BiologiQ, under the trade name ESR (“Eco Starch Resin”), contain PE and plasticized starch. They can be processed in the form of films, bags, articles processed by the injection method, or by thermoforming, etc. [22].

This paper analyzes some mixtures based on HDPE and plasticized starch. Since plasticized starch has poor mechanical properties [21], a small amount of plasticized starch was used to avoid the decrease of mechanical properties. The influence of some compatibilizers, a nanofiller—organo-montmorillonite (OMMT)—, and a small amount of ethylene propylene terpolymer rubber (EPDM) on the properties of the new materials was tested. This study can provide new information regarding the modification of the morphology, structure, thermal, and mechanical properties upon the introduction of small amounts of plasticized starch in HDPE. This study could be especially useful in terms of the reuse of post-consumer PE waste through mechanical recycling because, as we have presented, there are types of packaging based on PE and plasticized starch already on the market, and their sorting is very difficult.

## 2. Materials and Methods

### 2.1. Materials

To make the samples, TIPELIN 1100J high-density polyethylene from Mol Petrochemicals Co. Ltd., Tiszaújváros, Hungary, was used as the polymer matrix. Blends contain an amount of approx. 29% plasticized starch (PS), obtained from soluble potato starch from Lach-Ner, Czech Republic, plasticized with glycerin from Lach-Ner, Neratovice, Czech Republic, to which citric acid anhydrous from Reanal Laborvegyszer Kft., Budapest, Hungary, was added to improve compatibility. Polyethylene-graft-maleic anhydride (PE-g-MA) Admer NF 468E from Mitsui Chemicals Europe GmbH, Düsseldorf, Germany, was used as a compatibilizer between HDPE and PS. An elastomeric component was also used, namely, ethylene propylene diene terpolymer (EPDM) Nordel 4760 from Dow Chemical Company, Dupont Elastomer, Wilmington, DE, USA, a cross-linking agent: 1,3–1,4 bis(tert-butylperoxyisopropyl (peroxide), Luperox F 40 PE from Arkema, Colombes, France, and a cross-linking coagent, trimethylolpropane trimethacrylate (SR 350) ALCANPOUDRE TMPTMA-70 (TMPT) from SAFIC-ALCAN, Warrington, UK. Nanoclay Nanomer I.31 PS from Sigma–Aldrich, St. Louis, MI, USA, was used as nanofiller. Table 1 presents several characteristics of the materials used.

### 2.2. Obtaining Plasticized Starch

Plasticized starch (PS) was made by mixing starch (70 parts by weight) with glycerin (30 parts by weight) and citric acid (2 parts by weight) for 3–5 min in a Berzelius beaker. The mixing of the ingredients continued in the Plasti-Corder Brabender internal mixer 350E (Duisburg, Germany), where the gelatinization phenomenon took place and the thermoplastic/plasticized starch was obtained. The working parameters of the Plasti-Corder Brabender internal mixer were a temperature of 120–140 °C, a working time of 10 min, and a mixing speed of 30 revolutions/minute for the first 3 min and then 80 revolutions/minute for the next 7 min. In the first 3 min, the mixture heats up to the temperature of the mixing room, the starch granules hydrate/swell in the glycerin, and the viscosity of the mixture begins to increase, which is indicated by the increase in torque recorded by the Brabender mixer. In the next 7 min, when the rotational speed increases to 80 revolutions/minute, there is an increase in viscosity up to a maximum point (indicating the phenomenon of gelatinization) when a large number of starch granules break and stick together, followed by a decrease in the viscosity and torque (which may be due to the melting of some crystalline regions in the starch) [23], respectively, up to an equilibrium point, when the mixture is considered homogeneous.

### 2.3. Obtaining the Mixtures

The mixtures based on HDPE and plasticized starch were made in a Plasti-Corder Brabender internal mixer 350E (Duisburg, Germany) at 160 °C and the working time was 8 min. In the first 3 min, the rotational speed was 30 revolutions/min, the period during which HDPE melts and the ingredients are incorporated into the polymer matrix. In the next 5 min, the rotational speed increased to 80 revolutions/min because the aim was to achieve both a proper homogenization of the mixtures and the cross-linking of the copolymers in the HDPE melt under strong shearing forces (called the dynamic vulcanization method [24]), respectively, for intercalation and dispersion of the OMMT [25]. Table 2 shows the manufacturing recipes of the mixtures, expressed in grams.

### 2.4. Obtaining Test Specimens

The mixtures obtained from the mixer were processed using the compression method in order to obtain the test specimens needed to determine the characteristics. Frame-type molds and a Fortune Presses hydraulic laboratory press model no. TP 600, manufactured by Fontijne Grotnes, Vlaardingen, The Netherlands, were used. The working parameters were a pressing temperature of 170 °C and preheating for 5 min at contact pressure followed by compression molding for 3 min at the pressure of 5 MPa. The cooling was achieved up to a temperature of 40 °C, with a cooling time of 12 min, and pressure of 5 MPa. We obtained rectangular plates with dimensions of 150 × 150 mm^2^ and thicknesses of 2 and 4 mm, and plates of 50 × 50 mm^2^ with a thickness of 6 mm, respectively. From these, samples were made for testing, according to the respective method standards, using punch knives and an automatic punch (Figure S1). Before making the determinations, the samples were conditioned for 16 h at room temperature.

### 2.5. Laboratory Tests

The spectral data were recorded on a Nicolet iS50 FT-IR spectrophotometer (Thermo Fisher Scientific Inc., Madison, WI, USA) provided with an ATR investigation system. The scanning range was 4000 cm^−1^ to 400 cm^−1^ at a spectral resolution of 4 cm^−1^ and 32 scans.

The FTIR 2D maps were recorded with a Nicolet iN10 MX (Nicolet, Waltham, MA, USA) in the domain 4000–400 cm^−1^.

Thermal behavior was followed with a STA 449C F3 system, TG-DSC (thermogravimetry—differential scanning calorimetry) from Netzsch (NETZSCH-Gerätebau GmbH, Selb, Germany) between 20 and 900 °C in dynamic (50 mL/min) air atmosphere. The evolved gases were transferred through heated transfer lines and analyzed on the fly with the help of an FTIR Tensor 27 from Bruker (Bruker Co., Ettlingen, Germany) equipped with an internal thermostatic gas cell.

Scanning electron micrographs for analyzed samples were obtained with a QUANTA INSPECT F50 scanning electron microscope, FEI Company, Eindhoven, The Netherlands. In order to visualize how the plasticized starch was dispersed in the HDPE polymer matrix, cryo-fractured samples (at liquid nitrogen temperature) were immersed for 48 h in HCl 6N at 60 °C, as indicated in [26,27]. Samples were coated with gold to ensure conductivity.

Charpy impact strength was determined using a RAY-RAN device (energy range up to 25 J and speed range 2.8–3.8 m/s) according to ISO 179-1 [28] on type 1 rod-shaped test specimens, with sizes: length l = 80 ± 2 mm, width y = 10 ± 05 mm, thickness x = 4 ± 0.2 mm, with type A notch (R= 0.25 ± 0.05 mm, thickness under the notch x_k_ = 3.2 ± 0.2 mm), or unnotched. The pendulum hammer used had G= 1.189 kg, E= 5 J, and v= 2.9 m/s.

Vicat softening temperature was determined using a Heckert FWV R device, according to ISO 306 [29]—method A50—using a pressing weight of 5 kg and a heating rate of 50 °C/h.

Shore D hardness was determined according to ISO 868 [30], on 6 mm thick specimens using a Durotech model B202 durometer from SDL Atlas, Stockport, England. The tensile strength was tested according to ASTM-D638-14 [31], on type IV (dumbbell) specimens, with a thickness of 2 mm, at a test speed of 50 mm/min. The density of the samples was determined at room temperature according to ISO 1183-1 [32]. The determination of the action of liquids was carried out using samples in the shape of a disc with a radius of 8 mm and a thickness of about 2 mm. They were dried for 24 h in an oven at 50 °C, cooled in a desiccator, and then weighed (m1). The liquids in which the samples were immersed were water and toluene. The test temperature was room temperature and 80 °C for water, and for toluene—room temperature. The samples were placed in water or toluene for 72 h, then they were taken out, the excess liquid was wiped off, and they were weighed (m2). After drying at 50 °C in an oven for 24 h and cooling in a desiccator, the samples were weighed again (m3). Using the obtained values, the mass variation and liquid absorption were determined, using Equations (1) and (2):

(1) $$Mass\;variation\left(\%\right)=\frac{m2-m1}{m1}\times 100$$

(2) $$Liquid\;absorption\left(\%\right)=\frac{m2-m3}{m1}\times 100$$

Four test specimens were used for each determination and the arithmetic mean of determinations was calculated.

Melt flow index was determined according to ISO 1133 [33] at 205 °C with 2.16 kg load and a pre-heating time of 4 min. A specialized tester, Haake Melt Flow MT from Thermo Electron Corporation, Germany, was used. Three measurements were carried out to obtain each data point.

## 3. Results and Discussion

### 3.1. Scanning Electron Microscopy

**Figure 1:**
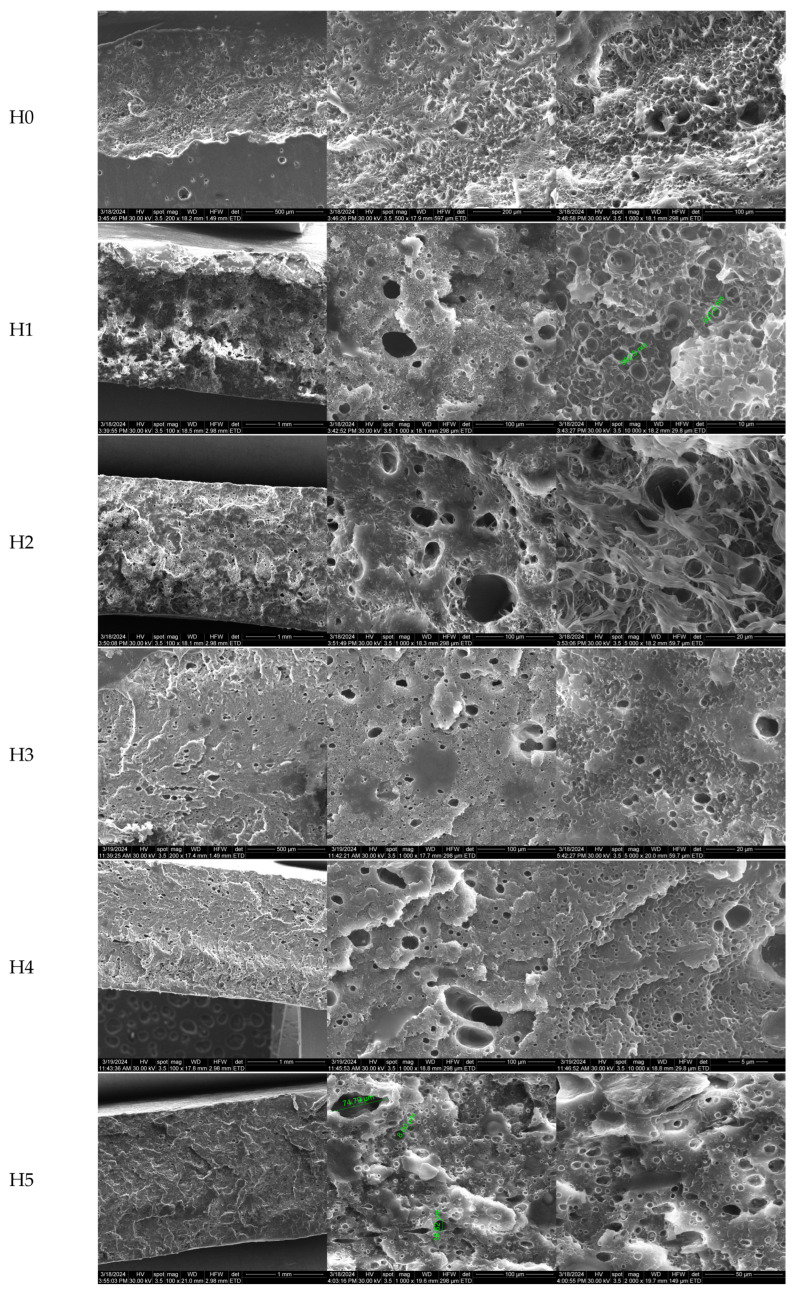
The SEM micrographs for the cryo-fractured cross-sections of H0–H5 samples, after 48 h treatment with HCl 6 N at 60 °C.

The components’ dispersion into the HDPE matrix is very important for composites and polymer blends as it determines the final mechanical properties. The plasticized starch is the minor component, and it can form droplets as a dispersed phase or large continuous domains in the major component (HDPE matrix). Cryo-fractured samples were treated with HCl 6 N to achieve the hydrolytic degradation of the starch and, using scanning electron microscopy, the created void pattern was exposed as contrast dark zones in the SEM micrographs from Figure 1.

The micrographs were acquired at low and high magnification to present the general aspect of the crack but also to indicate the details of the fracture.

The literature presents various morphologies for polymer blends, such as lamella, droplets, or fiber-like appearances [34,35]. It can be observed that all polymer blends exhibit round and oval-shaped pores, with sizes from micro- to nanometers, indicating a good dispersion of the starch in the HDPE matrix. The results are similar to those reported in [26]. The H0 sample presents a characteristic surface for plastic materials, with small imperfections generated by the fracturing process. In the H1 sample, the apparition of separate pores can be observed, indicating the previous presence of starch droplets. In general, the pores are well dispersed, without coalescence zones, which can be considered a homogeneity indicator. The micrographs obtained at higher magnification reveal the presence of a porous structure, with many pores under micrometer size, their size indicating better interactions among the components (Figure S2).

For the H2 sample, a smaller density of pores of all sizes is seen on the fracture surface, confirming the importance of the PE-g-MA compatibilizer. The highly homogenous structure indicates that the blending of polymers was improved by the compatibilizer, going further down to the polymer chain level. Therefore, some better mechanical properties are expected for the H2 blend vs the H1 sample.

A rather smoother surface was obtained for the H3 composition with EPDM, the addition of the rubber leading to the modification of the fracture aspect, with no fibrous or lamellar structures. The pores correspond to the removing of the starch from the fracture surface and have a similar dispersion to the one observed for the H2 sample (Figure S3).

The addition of the cross-linking agents in the H4 sample led to a different morphology of the fracture, with a smoother surface, and the presence of even smaller pores indicating a very good dispersion of the components into the HDPE matrix. Additionally, some lamellar structures can be observed on the surface, indicating a stiffer structure as a result of the formation of cross-linking bonds between the molecular chains that limit the movement. The presence of OMMT in the H5 composition led to similar results, with more visible fracture planes on the surface.

Some previous reports [36,37] indicate the obtaining of highly inhomogeneous mixtures when up to 20% starch was used together with polyethylene, with a large continuous starch phase. As can be observed, the pores for the H1–H5 samples are individual, without being interconnected and, more importantly, with no surface connection, indicating that the samples obtained can be considered stable, with low biodegradability as microorganisms do not have a continuous path from the surface into the mass of the samples. One critical factor for the biodegradability of a polymer blend is the phase continuity. As we have demonstrated, the starch is well dispersed, as droplets, from nano to micro size. As the surface of the samples did not present a porous structure, and the fractures indicated the lack of continuity of the starch phase, we can conclude that microbial colonization or moisture penetration cannot find a direct pathway. Therefore, such polymer blends will exhibit good stability in time, with the low biodegradability minimizing the microplastic formation.

### 3.2. Thermal Analyses

**Figure 2:**
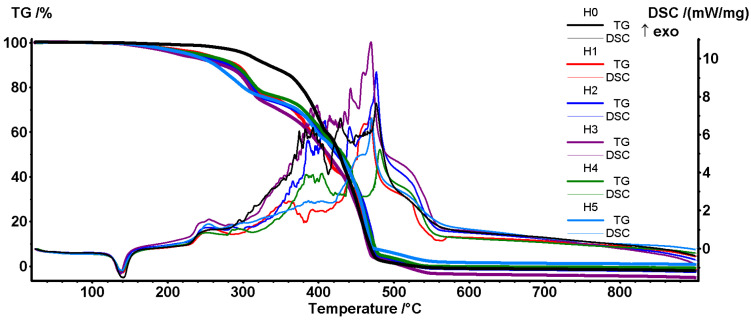
TG and DSC curves for H0–H5 polymeric blends.

The thermal analysis curves (TG and DSC) for all samples are presented in Figure 2, indicating a decrease in stability for all polymer blends compared with the H0 sample.

**Figure 3:**
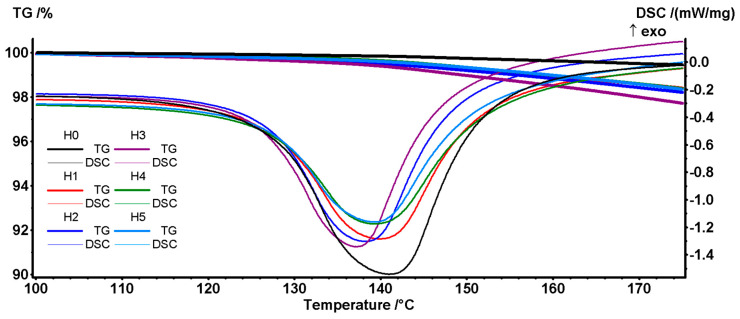
Detail of the TG and DSC curves for H0–H5 samples between 100–180 °C.

The H0–H5 samples melt ~126 °C and, while the pure H0 sample remains stable up to ~250 °C, the H1–H5 samples start to lose mass from 140 °C and up. This lower stability is generated by the presence of plasticized starch droplets [38] and becomes evident right after the melting process ends and the starch is exposed to the oxidative atmosphere (Figure 3).

The FTIR 3D diagrams for the evolved gases from the TG analysis of the H0 sample (Figure S4) and H5 sample (Figure S5) indicate that, for the H5 sample, the oxidation reactions start at slightly lower temperatures, as indicated by the point where CO_2_ and C-H fragments are detectable. Additionally, under 200 °C, the elimination of glycerin from plasticized starch is visible for the H5 sample, but not for the H0 sample.

The T1% values (the temperature at which the sample lost 1% of its initial mass) indicate a 50–60 °C difference between the H0 and the H1–H5 samples, with the sample H3 with EPDM exhibiting the lowest stability and also the lowest melting point.

At higher temperatures, the degradative–oxidative processes occur, as indicated by the rapid mass loss coupled with the strong exothermic effect. The multiple peaks on the DSC and DTG curves (Figure 2) indicate the existence of different oxidation reactions, partially overlapped, with the last shoulder corresponding to the burning of the residual carbonaceous mass.

The principal values obtained from thermal analysis are presented in Table 3.

The values for the crystallinity degree were calculated as the ratio between the experimental determined melting enthalpy (Table 3) and the theoretical melting enthalpy (293 J/g) of the 100% crystalline polymer reported in the literature [39], considering the HDPE content (%) of each sample. It can be observed that the crystallinity degree increased for all samples through the addition of plasticized starch, PE-g-MA, and EPDM. The presence of these macromolecules generates a short-range order for HDPE chains in their vicinity, as they act as nucleating agents. The addition of TMPT to the H4 sample led to a decrease in the crystallinity degree from 68.62 to 63.23%, but the further addition of OMMT did not influence this value in the H5 sample.

### 3.3. FTIR Spectroscopy and Microscopy

**Figure 4:**
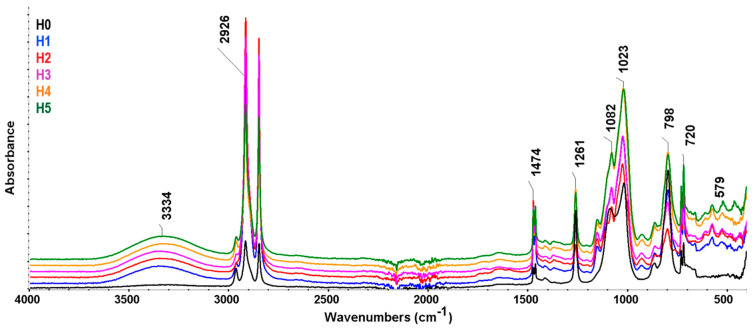
FTIR spectra for H0–H5 samples.

Figure 4 shows the FTIR spectra of samples. Strong absorption bands at 2925–2926 cm^−1^ and 2847–2848 cm^−1^ are attributed to the asymmetrical (ν_as_ CH_2_) and symmetrical (ν_s_ CH_2_) stretching vibrations of the methylene group, occurring both in HDPE and in plasticized starch.

The existence of double bands specific to aliphatic macromolecular compounds with a long linear chain can be noticed, namely, the double bands due to the deformation vibration of the methylene groups (-CH_2_-) from 1462 cm^−1^ and 1474 cm^−1^, namely, double absorption bands of the out-of-plane deformation vibration of C-H bonds at 730 cm^−1^ and 720 cm^−1^ [40]. Of these, the bands that appear at 1474 cm^−1^ and 730 cm^−1^ are attributed to the crystalline phase, and those at 1462 cm^−1^ and 720 cm^−1^ are attributed to the amorphous phase [26,41,42]. The band at 1258–1261 cm^−1^ may be due to the asymmetric bending vibration of the C-C-H or -CH_2_ twisting groups, and bands at 1078–1082 cm^−1^ may be due to C-C stretching vibrations [41] (Figure S6).

Wide absorption bands at 3300–3400 cm^−1^ are due to the -OH groups and association with intermolecular hydrogen bonds appearing in plasticized starch, respectively [43]. Bands at 1110–1170 cm^−1^ are specific to the stretching vibration of the C-OH bond existing in glycerin and starch. For samples containing plasticized starch, other changes are seen in the range 790–1000 cm^−1^ that can be attributed to the C–O–C group from starch [26,44]. The peak at 1150 cm^−1^ is attributed to the C–O stretch in the C–OH or C-O-C group in glycerin and starch [45,46]. Samples H4 and H5, containing cross-linking agents and OMMT, show an increase in the intensity of absorption bands situated around 1023 cm^−1^, 862 cm^−1^, and 798 cm^−1^, respectively, which may be attributed to the appearance of cross-links or indicate the existence of Si-C and Si-O bonds from OMMT. Sample H5 shows the appearance of a low-intensity absorption band at 464 cm^−1^ which may be due to the vibration absorptions of the Si−O−Si and Si−O−Al groups in the OMMT crystal lattice [47].

**Figure 5:**
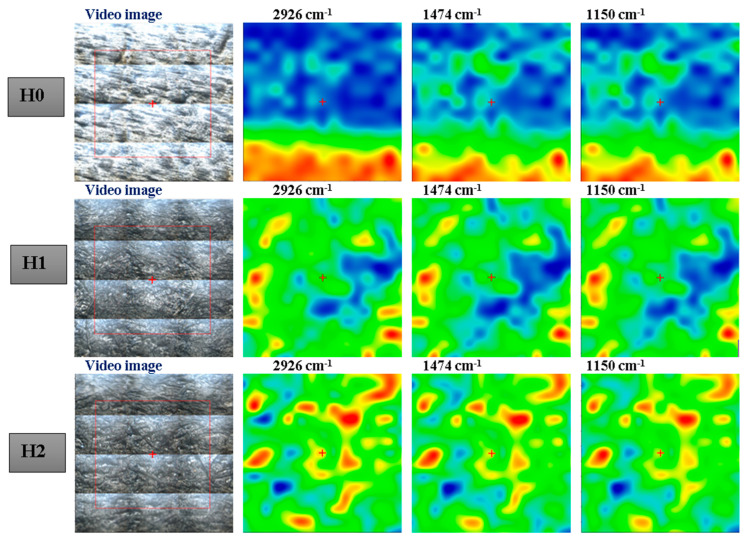

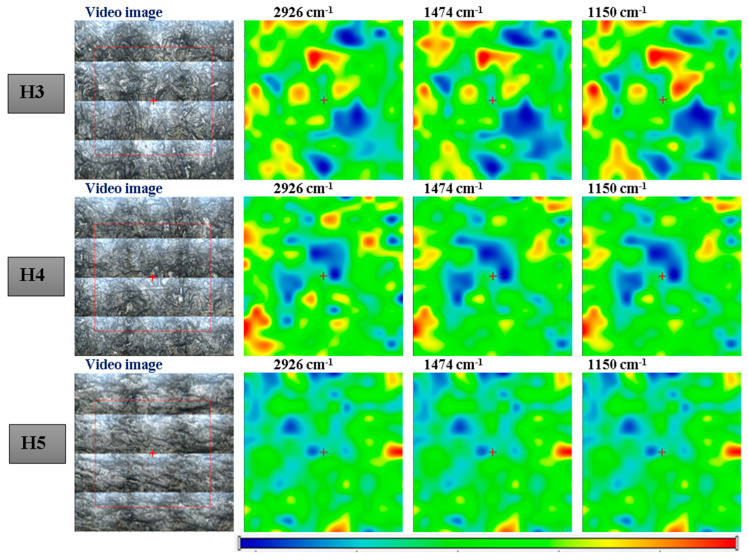
FTIR maps for H0–H5 samples.

For FTIR microscopy (Figure 5), we have chosen the 2D maps at three wavenumbers: 2931 cm^−1^ assigned to C-H stretching from CH_2_ moieties, 1474 cm^−1^ attributed to the deformation vibration of the methylene groups, and 1150 cm^−1^ assigned to the C–O stretch [48]. The blue areas indicate the lowest absorbance while the red areas correspond to the highest absorbance. As the FTIR maps for each sample are uniform, we can conclude that the polymer blends are homogenous, in good concordance with the SEM micrographs.

### 3.4. Physical-Mechanical Properties

The hardness expressed in Shore D degrees has high values (62–71° ShD), specific for hard materials, due to the high percentage of the crystalline phase existing in the HDPE polymer matrix. A decrease of approx. 6° ShD compared to the hardness of the control sample was observed when the plasticized starch was added, which could be due both to the plasticizer–glycerin, and to the decrease in the crystalline phase (although there is a higher degree of crystallinity for HDPE, the overall crystallinity of the sample decreases). The addition of EPDM rubber and the cross-linking agents or OMMT, respectively, led to a decrease of 1–2° ShD, which could be due to the increase in the percentage of the amorphous phase.

The tensile strength decreases by about 28% with the addition of plasticized starch (H1–H5), due to its weak mechanical properties or due to poor phase interaction. This aspect was also shown by other researchers [42,46], who showed that the tensile strength decreases significantly when a quantity of plasticized starch is added to the mixture. By using the PE-g-MA compatibilizer, there is an improvement in the tensile strength by 7.2%. According to the literature, chemical reactions can occur at the interface between the PE-g-MA groups and those existing in the plasticized starch, which can lead to an improvement in the compatibility of polyethylene with plasticized starch [26,49,50]. When adding the other ingredients (namely, EPDM, peroxide, TPMT, and OMMT), the tensile strength shows variations of 14.88–16.8 N/mm^2^. The mixture containing EPDM (sample H3) has a lower breaking strength by about 12.6% than sample H2 because it contains a larger amount of amorphous phase, thus reducing crystallinity and the mechanical properties, respectively [51]. Through the formation of cross-linking bonds between macromolecules in the mixture, as a result of the addition of small amounts of peroxide and cross-linking co-agent, an improvement in tensile strength by 12.9% is observed. This improvement in tensile strength is specific to materials where the elastomer is cross-linked in the melt of the thermoplastic material under high shear forces, using the method called dynamic vulcanization [52]. The addition of the OMMT nanofiller leads to a reduction of the overall crystalline phase and a 10.8% reduction in the tensile strength, which could be due to the reduction of the ductility of the sample.

The density increases from 0.99 g/cm^3^ (in the control sample H0) to 1.09 g/cm^3^ when adding PS, which has a higher density. A reduction in density from 1.09 to 1.08 g/cm^3^ can be observed in samples H4 and H5, which could indicate a more compact arrangement of the macromolecular chains as a result of the formation of some cross-linking bonds respectively obtaining a nanocomposite through the penetration of the polymer chains into OMMT galleries [52,53,54].

Melt flow index (MFI) at 205 °C with a load of 2.16 kg shows good values specific to HDPE, but decreases through the addition of plasticized starch (by 5.98%) and EPDM elastomer (by 12.5%) and shows a significant decrease (by 67.98–78.45%) in the dynamically cross-linked samples (H4–H5) as a result of the formation of cross-linking bonds between the molecular chains that limit the flow. Values for MFI and other physical-mechanical properties are presented in Table 4.

The Charpy impact strength of HDPE has high values, of 31.8 kJ/m^2^ on uncut samples, and 2.06 kJ/m^2^ on cut samples, respectively, but it drops significantly when plasticized starch is added (to 7.96 kJ/m^2^ and 1.08 kJ/m^2^, respectively). This aspect could be due both to the weak mechanical characteristics, specific to plasticized starch, and to a weak adhesion between the two polymer phases. The H2 sample, which contains a compatibilizer, and the H1 sample, show an improvement in this characteristic by 21.36% and 13.89%, respectively. A significant improvement of this characteristic is observed in samples containing EPDM, which may be due to the ability of EPDM rubber to absorb shocks. The dynamically vulcanized sample (H4) had a better impact resistance on cut specimens than that of the H3 sample (indicating the existence of stronger interfacial/cross-linking bonds) and the sample containing the nanofiller showed a reduction in impact strength because the reinforcement induced by OMMT led to an increase in the fragility of the samples [54].

The Vicat softening temperature has values of 101–104 °C and was influenced by the addition of plasticized starch and the compatibilizer. Similarly, the temperature range in which the melting of the samples was observed was 135–150 °C. It decreased by about 4 °C when plasticized starch (sample H1) was added and reached 135–136 °C for samples H4–H5, which presented a different morphology due to dynamic vulcanization and intercalation in the melt to obtain a nanocomposite [53,54].

The samples were tested for the action of liquids for 72 h. Table 5 shows the mass variation and liquid absorption after 72 h of immersion in water at room temperature and at 80 °C, and in toluene at room temperature, respectively. Since HDPE is hydrophobic and plasticized starch is hydrophilic [55], an increase in the mass variation after immersion in water at room temperature (from 0.27% to 1.33%) can be observed in sample H1 compared to sample H0, which could be due to the storage of some molecules of water at the interface between HDPE and plasticized starch. At 80 °C instead, there is a reduction in the mass variation from 1.17% for the control sample H0, to −2.23% for the H1 sample, which may indicate the dissolution of a quantity of PS in the water. At the same time, for sample H1, an increase in water absorption is observed to 5.37% at room temperature and to 9.89% at 80 °C, which confirms the penetration of an amount of water into the free cavities formed between HDPE and plasticized starch [55].

PE-g-MA addition leads to the improvement of interactions at the interface between HDPE and plasticized starch, and therefore a decrease to 0.96% of the mass variation for the H2 sample immersed in water at room temperature is observed. Nevertheless, when immersed in water at 80 °C, positive values of mass variation are obtained (5.30%), which may be due to the penetration of some water molecules into the cavities formed by dissolving some amounts of PS. At the same time, for sample H2, compared to sample H1, there is a reduction in water absorption to 1.39% at 23 °C and 4.31% at 80 °C, respectively.

The addition of EPDM to the sample H3 led to a minor increase in mass variation after immersion in water (from 0.96% to 0.99% at room temperature and from 5.30% to 7.12% at 80 °C). The amount of absorbed water also changed from 1.39% to 1.18% at room temperature and from 4.31% to 5.02% at 80 °C, respectively, most probably as a result of the increase in the percentage of the amorphous phase. When cross-linking agents were added to the sample H4, a mass variation of 0.82% at room temperature and 8.33% at 80 °C, respectively, was observed. By introducing a small amount of OMMT to the mixture (sample H5), an improvement in the behavior when immersed in water at a temperature of 80 °C was observed (the mass variation decreased at 4.63%), an aspect also reported by other researchers [56].

The variations of the water absorption values at room temperature and at 80 °C when adding the other ingredients (H2–H5 samples) are, in general, small.

The mass variation after 72 h immersion in toluene (Table 5) shows a different behavior than the one presented above, because toluene shows a high affinity towards HDPE and EPDM which is explained by the close values of the solubility parameters at room temperature [57,58]. Thus, although HDPE has a high degree of crystallinity, the mass variation values in toluene are 4.33% for the control sample. The mass variation for samples H1 and H2 (of 6.00%, and 5.53%, respectively) could be due to the change in the contact surface between HDPE and toluene as a result of changes in the attractive forces at the interface between HDPE and PS [55]. For samples H3–H5, an increase in the mass variation values was observed (from 6.22% to 7.42%) because the EPDM used contains a percentage of 90% amorphous phase and, therefore, the percentage of non-cross-linked EPDM could be dissolved in toluene at room temperature. We assume that the changes in the mass variation in samples H4 and H5, compared to sample H3, are explained by the decrease in the amount of EPDM dissolved in toluene as a result of the existence of some restrictions due to the cross-linking bonds as a result of the reinforcement with nanofiller [58]. The toluene absorption values showed the same trend and increased from 4.63% for the control sample to 7.89% for the H5 sample.

## 4. Conclusions

Some of the mechanical properties (tensile strength and Charpy impact strength) of the HDPE/plasticized starch mixture were improved by the addition of a compatibilizer (PE-g-MA) and other ingredients. These were influenced by the percentage of the overall crystalline phase, the cross-linking degree, the interface between the polymer matrix and the plasticized starch, the characteristics of the mixture components, etc. Thus, the thermal properties of the polymer blends were determined. The addition of plasticized starch led to a decrease in thermal stability after the melting process. The results of the scanning electron microscopy show that the plasticized starch is uniformly dispersed in the HDPE matrix, with the starch forming micrometer and nanometer individual droplets, without continuous domains. The behavior during immersion in water and in toluene, respectively, for 72 h, was good and was influenced primarily by the resistance of the polymer matrix to the action of the liquid in which the samples were immersed. This was influenced by the fact that the plasticized starch particles, as well as the other ingredients, are embedded in the HDPE polymer matrix and only a small amount of these interacted with the test liquid. The samples had hardness of 62–65° ShD, tensile strength values of 14.99–17.02 N/mm^2^, and melt flow indices of 1.81–10.7 g/10’ at 205 °C with 2.16 kg load. The new materials can be used for different applications such as packaging, construction, agriculture, or in the food industry. Additionally, the new materials have a smaller carbon footprint because part of the synthetic polymer (HDPE) has been replaced with a biodegradable polymer (starch) obtained from renewable resources.

## Figures and Tables

**Table 1:** Main characteristics of materials used.

Material	Characteristics
High-density polyethylene (HDPE) TIPELIN 1100J	Copolymer (with propene-1 as comonomer), melt mass-flow rate (190 °C, 2.16 kg) 8.0 g/10 min; density: 0.961 g/cm^3^; tensile stress at break: 15 MPa; hardness: 63° ShD, Vicat softening temperature: 128 °C.
Starch	Water insoluble substances 0.28%, loss on drying (105 °C) 17.52%
Glycerin	Acidity 0.02%, density 1.26 g/cm^3^
Citric acid anhydrous	C_8_H_8_O_7_, purity 99.8%, sulfate ash < 0.02%, chlorides (Cl) < 0.0005%, sulfates (SO_4_) < 0.02%, oxalates (C_2_O_4_) < 0.05%
PE-g-AM (Admer NF 468E)	Density: 0.92 g/cm^3^: melting point: 120 °C; hardness: 51° ShD; Vicat softening temperature: 95 °C; melt mass-flow rate (190 °C, 2.16 kg) 4.0 g/10 min
EPDM (Nordel 4760)	Containing 70 wt % ethylene and 4.9 wt % ethylenenorbornene (ENB), with a Mooney viscosity of 70 ML1 + 4 at 120 °C, density of 0.88 g/cm^3^, and crystallinity degree of 10%.
Luperox F 40 PE	40% assay, 3.8% oxygen active
TMPT (ALCANPOUDRE TMPTMA-70)	70% ingredient active and 30% precipitated silica
Nanoclay (Nanomer I.31 PS)	Powder, organo-montmorillonite (OMMT), contains 0.5–5 wt% aminopropyltriethoxysilan and 15–35 wt% octadecylamine, particle size ≤ 20 μm

**Table 2:** Manufacturing recipes.

Ingredients	Samples Symbol					
	H0	H1	H2	H3	H4	H5
HDPE, g	260	185	175	165	165	165
Plasticized starch, g	-	75	75	75	75	75
PE-g-MA, g	-	-	10	10	10	10
EPDM, g	-	-	-	10	10	10
Peroxide, g	-	-	-	-	0.2	0.2
TMPT, g	-	-	-	-	0.2	0.2
OMMT, g	-	-	-	-	-	7.5

**Table 3:** Principal numerical data from thermal analysis of H0–H5 samples.

Sample	T_1%_	T_5%_	T_10%_	Melting Onset	Melting Enthalpy	Crystallinity Degree (HDPE)
H0	209 °C	303 °C	333 °C	126.8 °C	157.4 J/g	53.72%
H1	156 °C	245 °C	291 °C	126.3 °C	126.2 J/g	60.53%
H2	153 °C	232 °C	283 °C	126.3 °C	123.8 J/g	62.77%
H3	148 °C	212 °C	271 °C	125.3 °C	127.6 J/g	68.62%
H4	159 °C	239 °C	288 °C	126.4 °C	117.4 J/g	63.23%
H5	159 °C	227 °C	263 °C	126.5 °C	114.7 J/g	63.56%

**Table 4:** Physical–mechanical properties and melt flow index.

Characteristics	H0	H1	H2	H3	H4	H5
Hardness, ° ShD	71.0 ± 0.9	65.0 ± 0.5	64.0 ± 0.5	64.0 ± 0.5	63.0 ± 0.5	62.0 ± 0.5
Tensile Strength, N/mm^2^	22.09 ± 3.03	15.87 ± 3.62	17.02 ± 1.22	14.88 ± 1.81	16.8 ± 0.58	14.99 ± 0.55
Density, g/cm^3^	0.99 ± 0.00	1.09 ± 0.00	1.09 ± 0.00	1.09 ± 0.00	1.08 ± 0.00	1.08 ± 0.00
Charpy Notched, kJ/m^2^ Charpy Unnotched, kJ/m^2^	2.06 ± 0.68	1.08 ± 0.24	1.23 ± 0.23	1.53 ± 0.23	2.27 ± 0.01	1.64 ± 0.22
	31.8 ± 5.67	7.96 ± 1.53	9.66 ± 2.75	20.29 ± 2.23	19.34 ± 6.32	14.63 ± 2.08
Softening Temperature VICAT, °C	104 ± 1	102 ± 1	101 ± 1	101 ± 1	101 ± 1	101 ± 2.5
Melting Range, °C	150 ± 5	144 ± 5	143 ± 5	144 ± 5	136 ± 5	135 ± 5
M.F.I	11.4 ± 0.04	10.7 ± 0.11	9.6 ± 0.11	8.4 ± 0.11	1.81 ± 0.86	2.69 ± 0.91

**Table 5:** Action of liquids—mass variation and absorption of liquids after 72 h water and toluene immersion.

Sample Code	Mass Variation After 72 h Water Immersion at 23 °C	Water Absorption After 72 h Immersion at 23 °C	Mass Variation After 72 h Water Immersion at La 80 °C	Water Absorption After 72 h Immersion at 80 °C	Mass Variation After 72 h Toluene Immersion at 23 °C	Toluene Absorption After 72 h Immersion at 23 °C
	%	%	%	%	%	%
H0	0.27 ± 0.16	0.39 ± 0.21	1.17 ± 0.08	0.81 ± 0.17	4.33 ± 0.93	4.63 ± 1.02
H1	1.33 ± 0.58	5.37 ± 0.59	−2.23 ± 0.75	9.89 ± 1.28	6.00 ± 0.17	7.11 ± 0.74
H2	0.96 ± 0.10	1.39 ± 0.15	5.30 ± 0.62	4.31 ± 0.99	5.53 ± 0.21	5.89 ± 0.31
H3	0.99 ± 0.13	1.18 ± 0.10	7.12 ± 0.23	5.02 ± 0.34	6.22 ± 1.52	6.64 ± 1.28
H4	0.82 ± 0.15	1.46 ± 0.16	8.33 ± 1.70	4.38 ± 2.92	6.73 ± 0.11	6.98 ± 0.30
H5	0.97 ± 0.15	1.66 ± 0.17	4.63 ± 0.89	4.09 ± 0.98	7.42 ± 0.22	7.89 ± 0.21

## Data Availability

The original contributions presented in the study are included in the article/supplementary material, further inquiries can be directed to the corresponding author.

## Supplementary Materials

The following supporting information can be downloaded at: https://www.mdpi.com/article/10.3390/polym16213051/s1, Figure S1: Picture of the H0-H5 samples (a) and close-up view (b); Figure S2: The SEM micrograph for H1 sample at 5000× magnification; Figure S3: The SEM micrograph for H3 sample at 5000× magnification; Figure S4: The FTIR 3D diagram for the H0 sample. The red line represents the FTIR spectrum at 330 °C and the green line is the evolving trace for the wavenumber 2933 cm^−1^ assigned to the C-H asymmetric vibration from –CH_2_ moieties; Figure S5: The FTIR 3D diagram for the H5 sample. The red line represents the FTIR spectrum at 320 °C and the green line is the evolving trace for the wavenumber 2933 cm^−1^ assigned to the C-H asymmetric vibration from –CH_2_ moieties; Figure S6: Detail of the FTIR spectra for H0-H5 samples.
