# Supplementary material for: Characterization of Mixtures Based on High-Density Polyethylene and Plasticized Starch
Source: Polymers (Basel). 2024 Oct 30;16(21):3051. doi: 10.3390/polym16213051 (PMC11548385; doi:10.3390/polym16213051)
Supplement: Supplementary file 1 [file polymers-16-03051-s001.zip › polymers-3260128-supplementary.pdf]

polymers

MDPI

Article

Characterization of Mixtures Based on High Density Polyethylene and Plasticized Starch

Maria Daniela Stelescu [1], Ovidiu-Cristian Oprea [2,3*], Doina Constantinescu [4], Ludmila Motelica [3,5], Anton Ficai [2,3], Roxana-Doina Trusca [2], Maria Sonmez [1], Dana Gurau [1], Mihai Georgescu [1], Rodica Roxana Constantinescu [1], Bogdan-Stefan Vasile [3,5], Denisa Ficai [2,3]

[1] Division Leather and Footwear Research Institute, National Research & Development Institute for Textiles and Leather, 93 Ion Minulescu St., 031215 Bucharest, Romania; dmstelescu@yahoo.com (M.D.S.); maria.sonmez@icpi.ro (M.S.); rodica.roxana@yahoo.com (R.R.C.); mihai.georgescu@icpi.ro (M.G.)
[2] Faculty of Chemical Engineering and Biotechnologies, National University of Science and Technology POLITEHNICA Bucharest, 1-7 Polizu St., 011061 Bucharest, Romania; ludmila.motelica@upb.ro (L.M.); anton.ficai@upb.ro (A.F.); denisa.ficai@upb.ro (D.F.)
[3] Academy of Romanian Scientists, 3 Ilfov St., 050044 Bucharest, Romania; vasile_bogdan_stefan@yahoo.com (B.S.V.)
[4] S.C. Monofil S.A, Uzinei, 1, Savinesti, Piatra Neamt, Romania, monofil.srl@gmail.com (D.C.)
[5] Research Center for Advanced Materials, Products and Processes, National University of Science and Technology POLITEHNICA Bucharest, 060042 Bucharest, Romania
* Correspondence: ovidiu.oprea@upb.ro

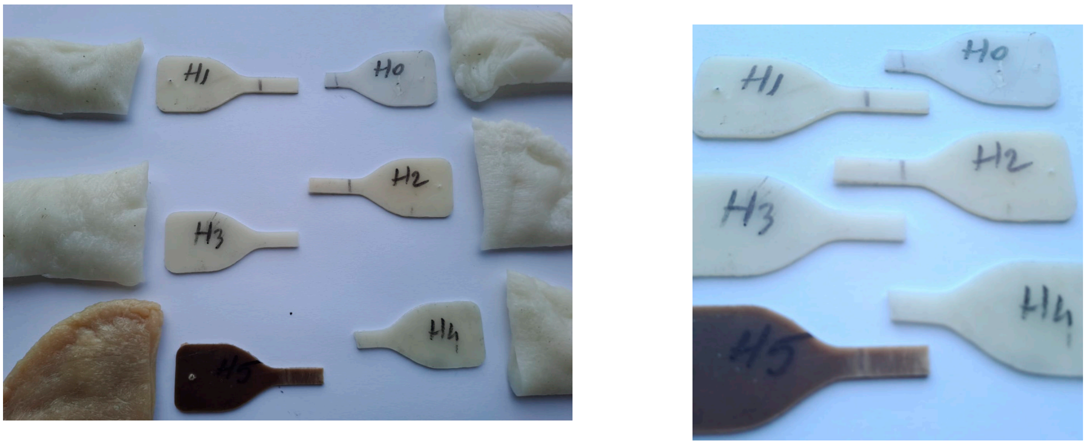

a. b.

Figure S1. Picture of the H0-H5 samples (a) and close-up view (b).

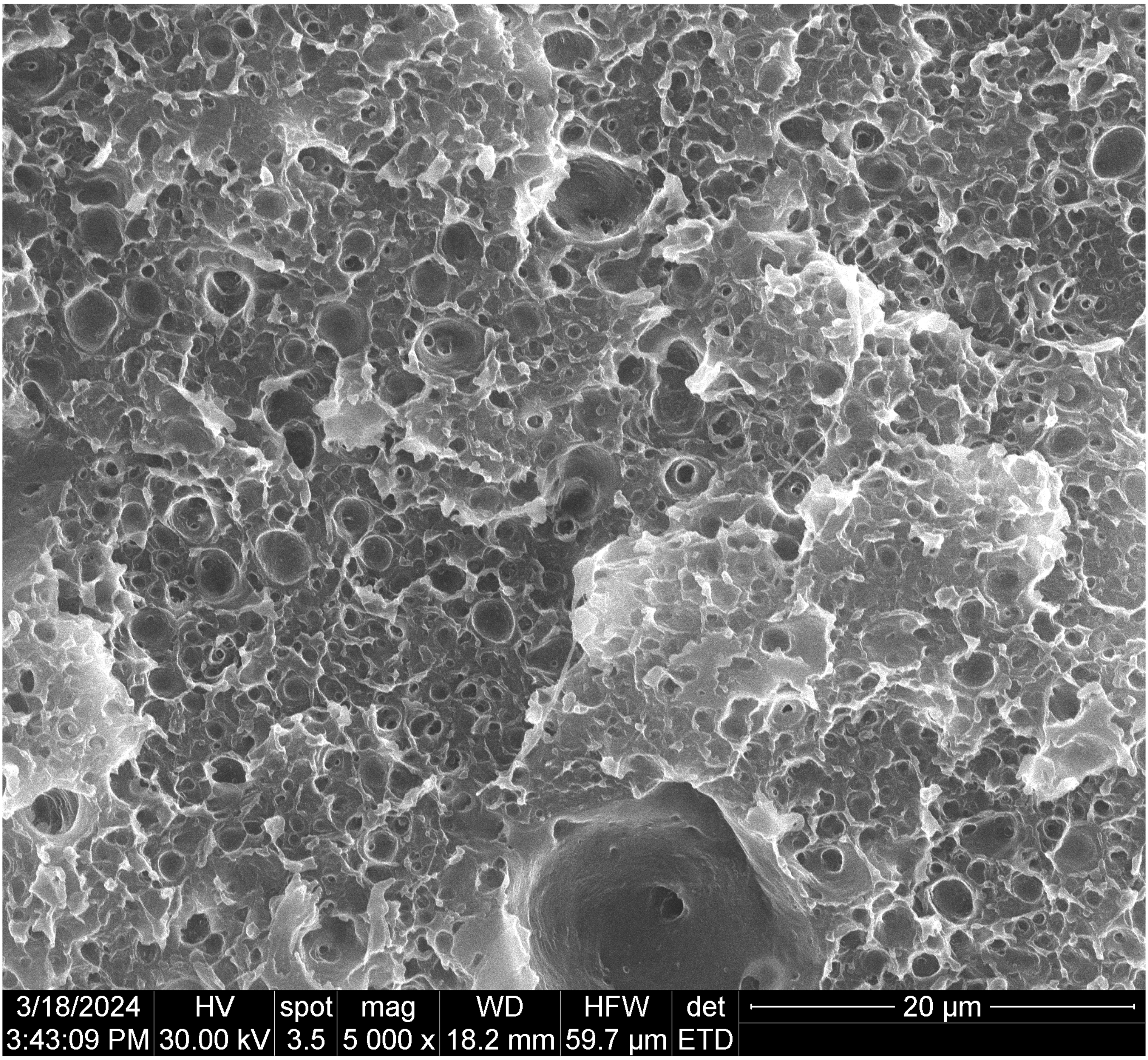

Figure S2. The SEM micrograph for H1 sample at 5000x magnification.

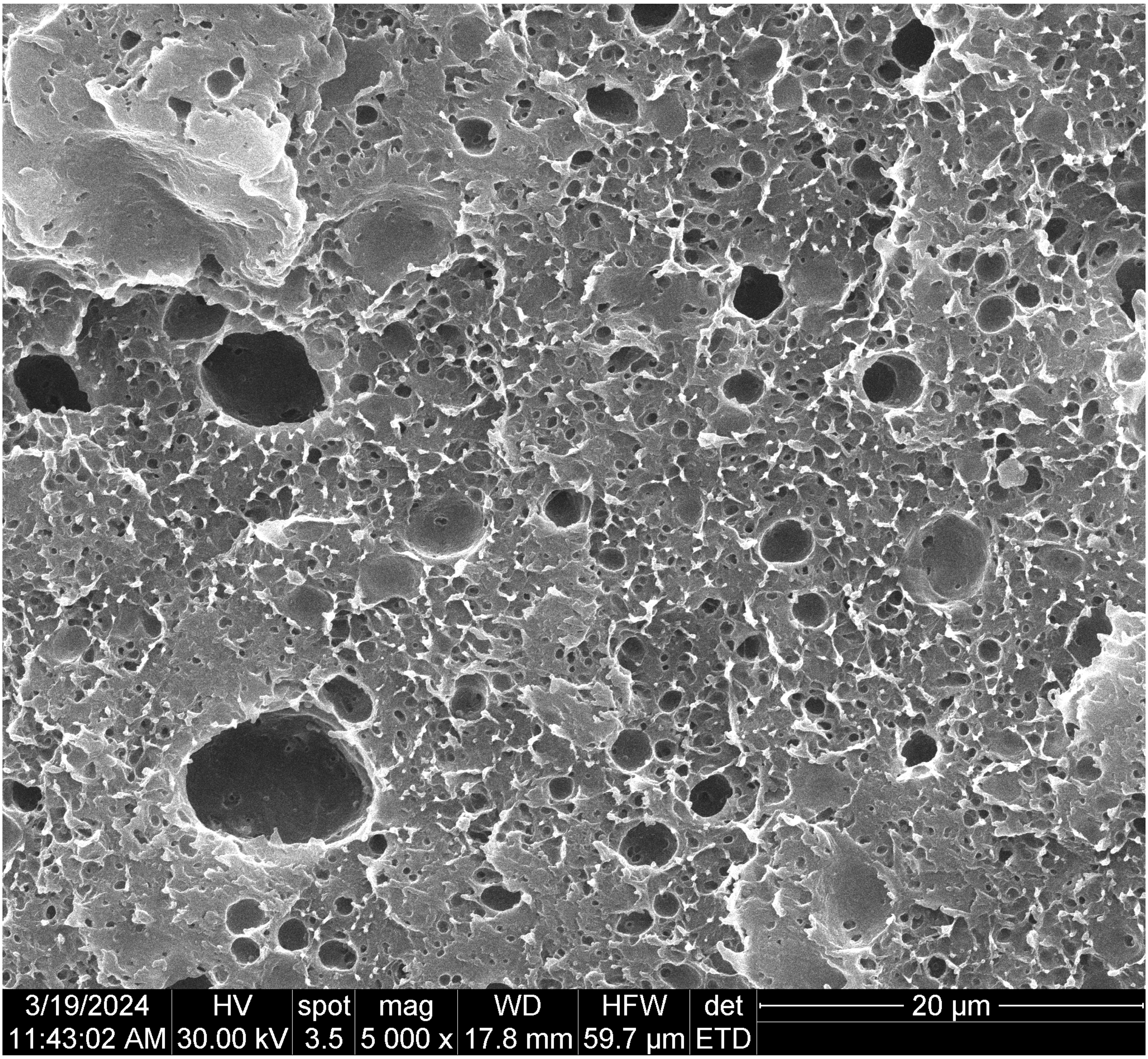

Figure S3. The SEM micrograph for H3 sample at 5000x magnification.

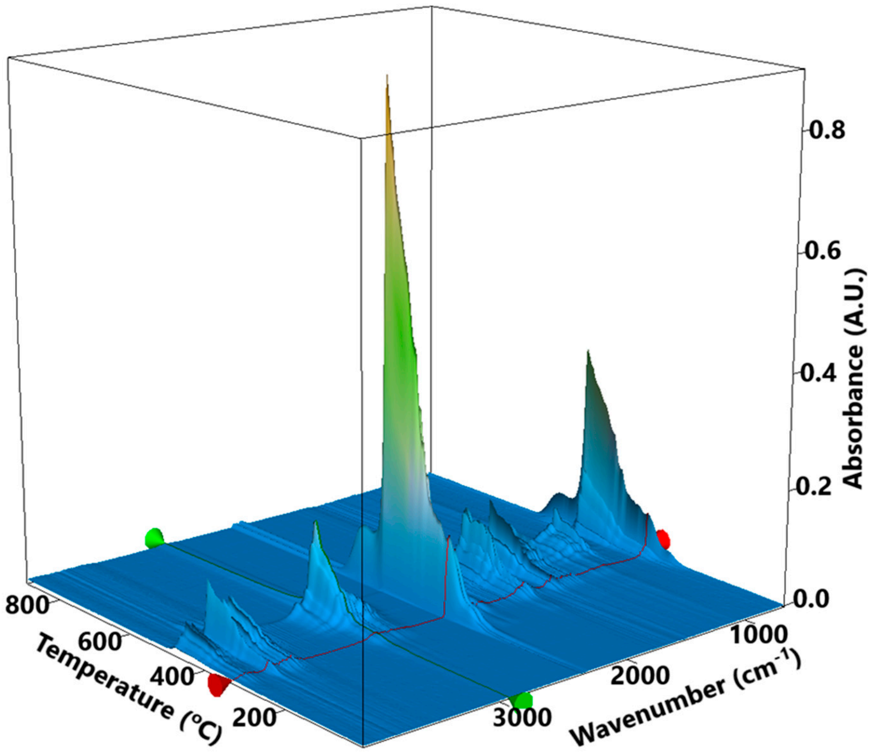

Figure S4. The FTIR 3D diagram for the H0 sample. The red line represents the FTIR spectrum at 330°C and the green line is the evolving trace for the wavenumber 2933 cm^{-1} assigned to the C-H asymmetric vibration from –CH2 moieties.

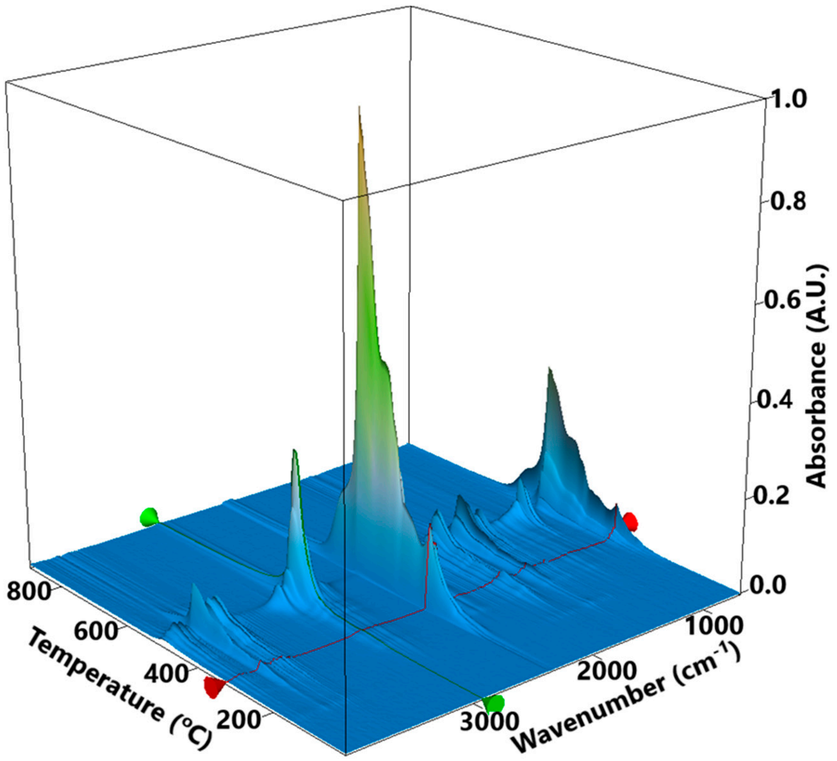

Figure S5. The FTIR 3D diagram for the H5 sample. The red line represents the FTIR spectrum at 320°C and the green line is the evolving trace for the wavenumber 2933 cm^{-1} assigned to the C-H asymmetric vibration from –CH2 moieties.

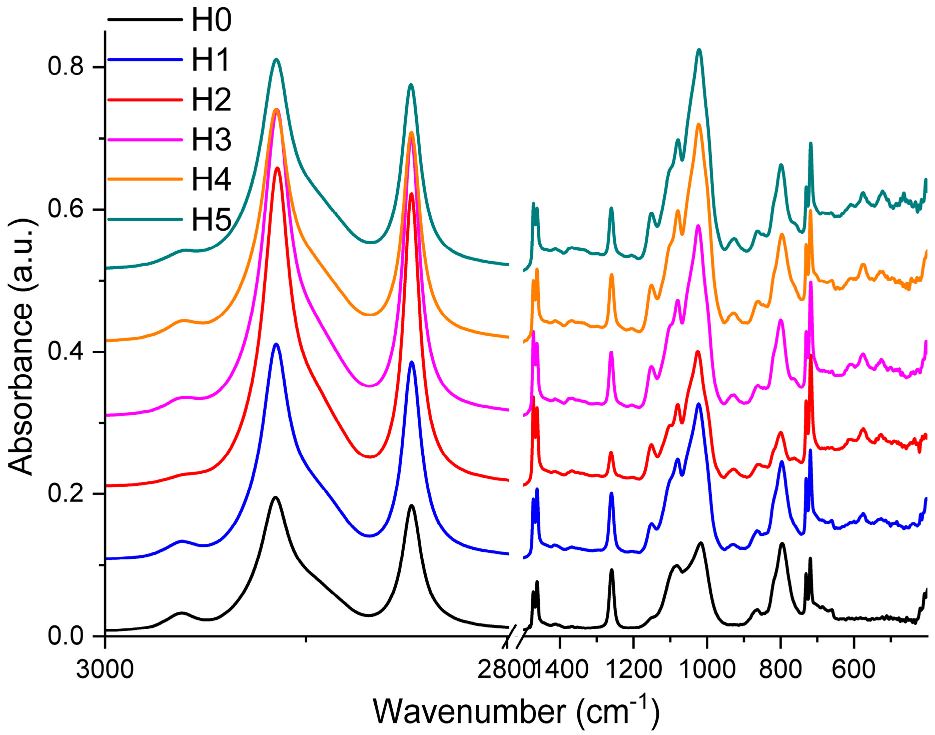

Figure S6. Detail of the FTIR spectra for H0-H5 samples.
